# Retraction Note: Neuroinflammation is associated with infiltration of T cells in Lewy body disease and α-synuclein transgenic models

**DOI:** 10.1186/s12974-026-04011-x

**Published:** 2026-08-22

**Authors:** Michiyo Iba, Changyoun Kim, Michelle Sallin, Somin Kwon, Anjali Verma, Cassia Overk, Robert A. Rissman, Ranjan Sen, Jyoti Misra Sen, Eliezer Masliah

**Affiliations:** 1 Laboratory of Neurogenetics, Molecular Neuropathology Section, National Institute on Aging, National Institutes of Health, Bethesda, MD 20892 USA; 2 Laboratory of Clinical Investigation, Immune Cells and Inflammation Section, National Institute on Aging, National Institutes of Health, Baltimore, MD 21224 USA; 3 Department of Neurosciences, University of California, La Jolla, San Diego, CA 92093 USA; 4 Laboratory of Molecular Biology and Immunology, Gene Regulation Section, Biomedical Research Center, National Institute on Aging, National Institutes of Health, Baltimore, MD 21224 USA; 5 Department of Medicine, The Johns Hopkins University School of Medicine, Baltimore, MD 21287 USA; 6 Division of Neuroscience, National Institute on Aging, National Institutes of Health, Bethesda, MD 20814 USA

**Retraction Note: J Neuroinflamm 17, 214 (2020)**

**https://doi.org/10.1186/s12974-020-01888-0**

The Editors-in-Chief have retracted this article. After publication, concerns were raised regarding some of the data presented in the figures, specifically:

- Discrepancies in Fig. 3A between the single-antibody and merged images;
- Fig. 4A Neocortex DLB images appear to contain a duplicated area;
- Fig. 7A α-syn tg Neocortex images appear to contain a duplicated area.

The Editors-in-Chief therefore no longer have confidence in the presented data.

Elezier Masliah does not agree with this retraction. The other authors have not responded to any correspondence from the editor or publisher about this retraction.

